# Medical students’ intention to integrate digital health into their medical practice: A pre-peri COVID-19 survey study in Canada

**DOI:** 10.1177/20552076221114195

**Published:** 2022-07-21

**Authors:** Guy Paré, Louis Raymond, Marie-Pascale Pomey, Geneviève Grégoire, Alexandre Castonguay, Antoine Grenier Ouimet

**Affiliations:** 1 10014 HEC Montréal, Montreal, Canada; 2 14847 Université du Québec à Trois-Rivières, Trois-Rivieres, Canada; 3 5622 Université de Montréal, Montreal, Canada; 4 4257 HEC Montréal and Queen’s University, Kingston, Canada

**Keywords:** Digital health, eHealth, medical education, medical practice, artificial intelligence, COVID-19, survey

## Abstract

**Objective:**

We aimed to explore the factors that influence medical students’ intention to integrate dHealth technologies in their practice and analyze the influence of the COVID-19 pandemic on their perceptions and intention.

**Methods:**

We conducted a two-phased survey study at the University of Montreal's medical school in Canada. The study population consisted of 1367 medical students. The survey questionnaire was administered in two phases, that is, an initial survey (t_0_) in February 2020, before the Covid-19 pandemic, and a replication survey (t_1_) in January 2021, during the pandemic. Component-based structural equation modeling (SEM) was used to test seven research hypotheses.

**Results:**

A total of 184 students responded to the survey at t_0_ (13%), whereas 138 responded to the survey at t_1_ (10%). Findings reveal that students, especially those who are in their preclinical years, had little occasion to experiment with dHealth technologies during their degree. This lack of exposure may explain why a vast majority felt that dHealth should be integrated into medical education. Most respondents declared an intention to integrate dHealth, including AI-based tools, into their future medical practice. One of the most salient differences observed between t_0_ and t_1_ brings telemedicine to the forefront of medical education. SEM results confirm the explanatory power of the proposed research model.

**Conclusions:**

The present study unveils the specific dHealth technologies that could be integrated into existing medical curricula. Formal training would increase students’ competencies with these technologies which, in turn, could ease their adoption and effective use in their practice.

## Introduction

Digital health (dHealth), which is defined by Fatehi *et al*.^1^ as “the proper use of technology for improving the health and wellbeing of people at individual and population levels, as well as enhancing the care of patients through intelligent processing of clinical and genetic data” (p.71), has attracted lots of attention in the past decade worldwide. Every day, hundreds of dHealth innovations, technologies, and smart devices are released at lightning speeds,^2^ which has amplified into a US $84 billion world industry with a projected increase to over US $220 billion by 2026.^1^ Health Canada and the Food and Drug Administration (FDA) consider a broad spectrum of technologies under the concept of digital health; including mobile health applications, wireless medical devices, telehealth and telemedicine, clinical information systems, big data and artificial intelligence (AI), and robotics, to name a few.^2^

The effective use of dHealth technologies by clinicians, managers, policy makers and patients has never been more relevant than it is today. Previous research has shown that dHealth has the potential to prevent disease^3,4^ and lower healthcare costs,^5,6^ while helping patients monitor and self-manage chronic conditions^7,8^ or optimizing the identification and use of available community resources by frail and isolated older adults.^9,10^ It has also been shown that dHealth technologies can improve the quality, continuity, and availability of care,^11–13^ tailor medical treatments for individual patients^14,15^ and facilitate remote patient assessment and follow-up during pandemics.^16,17^

For the practice of medicine to continue transforming itself and producing better outcomes for patients, future generations of physicians must be able to navigate with ease in an ever-changing digital environment.^18^ To this end, the literature teaches us that the training of medical students is an important factor in the adoption of dHealth technologies and that the interaction of future physicians with such technologies during their training allows the development of basic patient care competencies as well as dHealth knowledge, self-efficacy, and diagnostic accuracy.^19–23^ Yet, it is recognized that too many medical schools integrate dHealth little or not at all into the formal training of future physicians, which poorly prepares them for the changing reality of clinical practice.^24–26^ This underexposure causes medical students to have limited understanding of dHealth^27^ and is prone to lead to bad behaviors in medical students (eg, violation of patient confidentiality), which can ultimately lead to severe legal consequences.^28^

While several studies have identified important barriers and facilitators to the development of dHealth education in the medical curriculum^29,30^ and it appears important to accelerate this integration,^31^ we know very little about the perspectives of medical students on this issue. Indeed, very few studies investigated medical students’ beliefs about, familiarity with, and intention to integrate dHealth. Moreover, most existing studies focus on a particular type of dHealth technologies, leaving a more comprehensive picture unaccounted for. For instance, Sit *et al*.^32^ explored the attitudes of 484 United Kingdom (UK) medical students regarding training in AI technologies, their understanding of AI, and career intention towards radiology. Findings reveal that medical students do not feel adequately prepared to work alongside AI but understand the increasing importance of AI in healthcare and would like to receive more training on the subject. As another example, Yaghobian *et al*.^33^ conducted a national study on telemedicine training by 3312 medical students and residents in France. Positive attitudes towards telemedicine, particularly in relation to improving patients’ access to care, were observed. However, the majority of respondents felt they were not trained enough and would like to see training in telemedicine increase. Similar results were found in another study published in the United States, where medical students acknowledged the relevance of and need for telemedicine education in the curriculum.^34^

In light of the above, little empirical knowledge is available on medical students’ views on, experimentation with, and intention to integrate dHealth technologies. Further, prior studies either focused on a specific dHealth technology [eg,^35^^]^ or provided a vague definition of dHealth.^36^ Importantly, prior studies soliciting medical students’ opinions were conducted prior to the COVID-19 pandemic. In order to explore the factors that influence medical students’ intention to integrate dHealth technologies in their medical practice and analyze the influence of the COVID-19 pandemic on their perceptions and intention related to dHealth, we conducted a two-phased survey of medical students in Canada. Precisely, our study aims to answer the following research questions: *Do medical students intend to integrate dHealth into their future medical practice? What factors influence medical students’ intention? Did COVID-19 influence medical students’ perceptions and intention related to dHealth?* Given the present dearth of knowledge on these questions and their increased relevance in the context of the ongoing pandemic,^37^ the present study's intended contribution to research and practice lies in a clear and concise characterization and explanation of the role of dHealth in medical education.

### Theoretical model

To address the above-mentioned research questions, we first developed a theoretical model. One of the main theoretical foundations of this study is Triandis’ theory of interpersonal behavior^38^ which posits that individuals’ behavioral intention is influenced by their beliefs toward the behavior. Triandis defines beliefs as assessments of what an individual thinks about the object of interest There is no implied goodness or badness in beliefs, but only an assessment of what one thinks exists or does not exist For instance, an individual may hold a belief that computers in general contribute to the improvement of society. In this study, medical students’ beliefs were operationalized regarding AI-related technologies specifically rather than dHealth technologies generally, as AI-based tools have the most potential to fundamentally alter and significantly improve the practice of medicine.^23,39,40^ We posit that the stronger the medical students’ beliefs that AI can positively impact the medical profession as well as their own medical practice, the greater their intention to integrate dHealth technologies (including AI tools) into their future practice.

Second, we postulate that perceived facilitating conditions are another critical determinant of medical students’ behavioral intention related to dHealth adoption. Originating in the technology acceptance model (TAM), a theory that models how users come to adopt a new technology,^41^ facilitating conditions are external factors that influence an individual's perceptions of the difficulty with which a task (eg, use of dHealth technologies) may be performed.^42–46^ In the present study, facilitating conditions are operationalized as students’ level of exposure to or experimentation with dHealth technologies during their medical education. We posit that the more medical students experiment with dHealth technologies during their degree, the higher their intention to integrate dHealth in their medical practice.

Third, our theoretical model includes perceived usefulness, another TAM variable, which is defined as the degree to which a person believes that using a particular technology or information system would enhance their job performance.^41^ Adapted to the present study context, perceived usefulness refers to medical students’ perceptions of the importance and relevance of integrating dHealth into the medical curriculum. We posit that the more one thinks that all students should receive training in dHealth as part of their medical degree, the more their intention to integrate dHealth in their own medical practice.

Last, following prior research on dHealth training [eg,^18,32,47^] as well as various studies testing the TAM [eg,^42–44^] another construct, called individual background, was added to our research model. This construct was operationalized as a composite of three individual characteristics: gender, age, and academic level. Due to the exploratory nature of this study, we simply assert that students’ individual background is likely to influence their views and perceptions on dHealth technologies.

**Figure 1.**
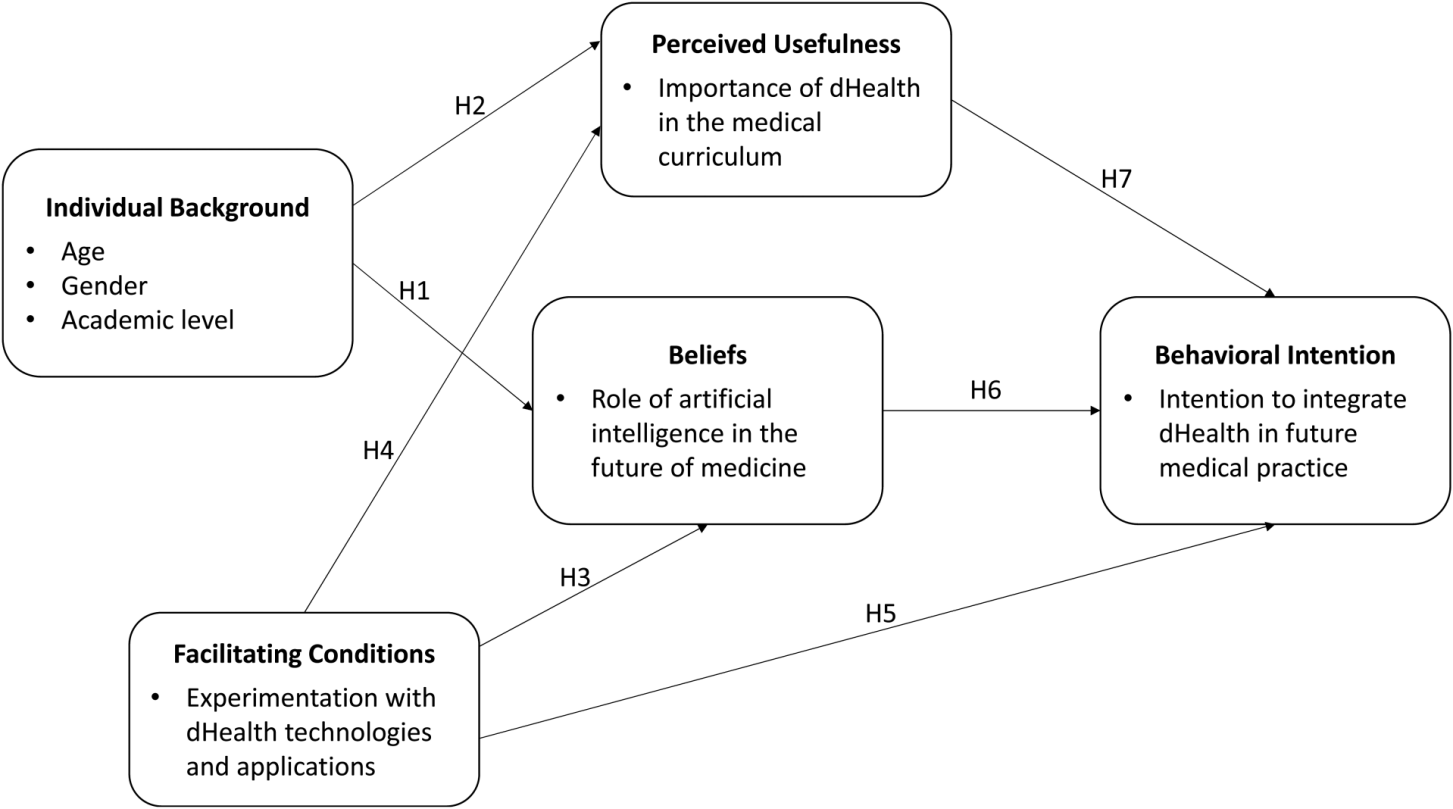
Theoretical model.

The theoretical model to be empirically tested in this study is shown in Figure 1. Given the relationships postulated by the theoretical lens mobilized above as well as the empirical relationships identified in the extant literature, seven hypotheses were formulated as summarized in Table 1. The first two hypotheses emanate from the TAM wherein medical students’ individual background is deemed to influence their beliefs about the role of dHealth technologies (more particularly AI) in medicine and their perceived dHealth education needs. For their part, hypotheses 3, 4 and 5 also emanate from the TAM wherein external facilitating conditions are deemed to influence beliefs toward a behavior, perceived usefulness, and behavioral intention. Hypothesis 6 is derived from the theory of behavior proposed by Triandis and states that medical students’ beliefs are deemed to positively influence their behavioral intention. The final hypothesis also originates from the TAM and posits a positive relationship between perceived usefulness and behavioral intention.

**Table 1.** Research hypotheses.

#	Hypotheses
1	Medical students’ individual background will likely influence their beliefs about the role of AI in the future of medicine
2	Medical students’ individual background will likely influence their perceptions relating to the importance of integrating dHealth into medical education
3	The greater the students’ experimentation with dHealth during their medical education, the stronger and more positive their beliefs about the role of AI in the future of medicine
4	The greater the students’ experimentation with dHealth technologies during their medical degree, the greater their perceived dHealth education needs
5	The greater the students’ experimentation with dHealth technologies during their medical education, the greater their intention to integrate dHealth into their medical practice
6	The stronger the medical students’ beliefs about the positive impact of AI on medicine, the greater their intention to integrate dHealth into their medical practice
7	The stronger the medical students’ perceptions related to the inclusion of dHealth into the medical curriculum, the greater their intention to integrate dHealth into their medical practice

## Methods

### Setting and data collection

The present study was conducted at the University of Montreal's (UM) medical school in Canada. During the 5-year long undergraduate medical curriculum, no formal dHealth education or training is provided to students. However, students have access to the EDUlib online training platform which offers educational content on a variety of subjects including health and information technologies, as well as to symposia and conferences on different aspects of dHealth. Also, workshops on mobile health and EMR systems are mandatory for students in their clerkship, a stage at which students are called upon to experiment with different dHealth technologies. Finally, issues related to the use of social media, email, and mobile applications by healthcare professionals are also covered in elective seminars.

The study population consisted of 1367 UM medical students. The survey questionnaire was administered in two phases, that is, an initial survey (t_0_) in February 2020, before the Covid-19 pandemic, and a replication survey (t_1_) in January 2021, during the pandemic. It was distributed to all students via the medical school's mailing list The study was also promoted via social media groups that are only accessible by UM medical students and the local medical student association sponsored the survey via its newsletter. There was no incentive for students to fill out the online questionnaire and there were no negative consequences if students did not participate.

The invitation contained a hyperlink directing the participants to the questionnaire through a secure Web site. The online questionnaire was developed on the Qualtrics survey platform. Qualtrics complies with applicable data privacy laws in its role as a data processor of customer data, as indicated on the company's website.

### Questionnaire development

We were unable to locate any pre-existing questionnaire that assessed the variables included in our research model; hence, we decided to develop our own instrument. The survey design underwent several rounds of iteration, and final validation was performed with a group of 10 UM medical students who were excluded from the sampling population. The final survey instrument consisted of 70 5-point Likert questions and 8 yes/no questions. The four tables located in Appendix I provide essential information on the questionnaire's content and on measurement items and scales.

### Statistical analysis

The data were first analyzed through descriptive statistics (mean, standard deviation, percentage) and further examined through analyses of variance and principal component analyses, using the IBM SPSS software v28. Component-based structural equation modeling (SEM) was then used to test the research hypotheses (cf. Figure 1). As implemented in the SmartPLS software, the partial least squares (PLS) technique was chosen for its robustness with regard to the distribution of residuals and its greater affinity for exploratory rather than confirmatory research purposes when compared to covariance-based SEM techniques such as AMOS and EQS.^48^

### Ethics approvals

The survey questionnaire was approved by the UM's ethics committee on October 29, 2019 (#CERSES-19-108-D). Informed consent was obtained from all participants. All methods were carried out in accordance with relevant guidelines and regulations.

## Results

### Demographics

A total of 184 students responded to the initial survey at t_0_ (13%), whereas 138 responded to the replication survey at t_1_ (10%). As shown in Table 2, most participants were female (65% at t_0_ and 70% at t_1_). The mean age was 23 years, which is comparable to the average age of medical students at UM. While our sample was evenly distributed between the first and fifth year of medical studies at t_0_, there were fewer fourth- and fifth-year medical students at t_1_.

**Table 2.** Profile of the respondents.

Medical students’ background		Pre-COVID-19		Peri-COVID-19	
		t_0_ (n = 184)		t_1_ (n = 138)	
		N	%	N	%
Academic level	Preparatory year	40	22%	28	20%
	First year preclinical	36	20%	32	23%
	Second year preclinical	43	23%	55	40%
	First year clerkship	33	19%	8	6%
	Second year clerkship	32	17%	14	10%
Gender	Female	119	65%	92	70%
	Male	65	35%	40	30%
Age	Mean	22.9		22.6	
	Standard deviation	3.5		2.7	
	Minimum	18		18	
	Maximum	38		35	

### Descriptive analysis

For analytical purposes, individual dHealth technologies and applications were grouped under five technology “bundles” that were named basic IT systems (eg, electronic medical records, clinical information systems), advanced dHealth (eg, robotics, virtual reality), telehealth (eg, teleconsultation, tele-expertise), AI-related technologies (eg, artificial intelligence, machine learning) and mobile applications (eg, UpToDate, BMJBestPractice).

First, with regard to the questions relating to experimentation with dHealth technologies, a large majority of the medical students had little occasion to experiment with advanced dHealth, telehealth, and AI-related technologies in the course of their medical curriculum (see Table A1 in Appendix I). A minority of the participants reported having been somewhat or very exposed to basic IT systems (i.e. EMR, CIS, and iEHR systems) and two specific mobile apps, namely, UpToDate and the Quebec Health Technology Assessment (HTA) Institute's mobile app.^3^

Second, a vast majority of respondents agreed that all students should receive formal dHealth education as part of their medical degree. As shown in Table A2 in Appendix I, medical students believe that the most important dHealth education needs concern, by order of importance, basic IT systems, telehealth, AI-related technologies, and robotics. A majority of respondents, however, perceive lesser need concerning other advanced dHealth technologies including blockchain, internet of things, virtual reality, and augmented reality.

Third, a large majority of the sampled students at both t_0_ and t_1_ are observed to have strong beliefs toward AI-related technologies, be it in terms of these technologies’ impact on the medical profession, in general, as well as on many medical specialties such as radiology and anatomopathology (see Table A3 in Appendix I). Moreover, most students indicate that they expect to use AI-based tools in support of one or more of their future medical activities including image and data analysis, diagnosis and, to a lesser extent, prognosis.

Fourth, a large majority of students declared an intention to integrate dHealth into their medical practice. As shown in Table A4 in Appendix I, this intention is most important with regard to disease prevention, diagnosis, and treatment, followed by patient communication and consultation, and patient monitoring and follow-up activities.

Last, when comparing the variable means between the two samples (t_0_ and t_1_), as presented in Table 3, *t*-test analyses confirm statistically significant differences (*P* < 0.001) on a few research variables. For one thing, medical students in the replication study (t_1_), on average, experimented more with telehealth technologies and, conversely, experimented less with basic IT systems and mobile applications than those in the initial survey (t_0_). This last result may be due to the fewer proportion of fourth- and fifth-year students who participated at t_1_ in comparison to t_0_. Further, medical students surveyed at t_1_ expressed a greater need for education on telehealth technologies than those surveyed at t_0_. This specific need was ranked first by students in the replication study, whereas it was ranked second by participants in the initial study. Here, one may tentatively explain these differences by the advent of the COVID-19 pandemic, which has brought telemedicine to the forefront of medical training and medical practice.^49^

**Table 3.** Comparison of medical students’ views and intention between t_0_ and t_1_.

Research construct	Pre-COVID-19	Peri-COVID-19	T-test
Research variable	(n = 184)	(n = 138)	(two-tailed)
	mean	mean	
Individual Background			
Age	22.9	22.6	1
Gender	0.65	0.7	-0.9
Academic level	2.9	2.6	1.9
Experimentation with dHealth technologies			
Basic IT systems	1.8	1.4	4.2***
Advanced dHealth	1.2	1.1	2.4**
Telehealth	1.2	1.5	−4.6***
AI-related technologies	1.3	1.2	2
Mobile applications	1.5	1.3	3.6***
Importance of dHealth in medical curriculum			
Basic IT systems	4.1	4.1	0.3
Advanced dHealth	3.4	3.4	-0.1
Telehealth	3.7	4.1	−5.0***
AI-related technologies	3.5	3.5	0.3
Beliefs about impact of AI-related technologies			
On the medical profession	3.6	3.5	1.2
On various medical specialties	3.4	3.3	0.8
On one's own medical practice	3.9	3.6	0.8
Intent to integrate dHealth in medical practice			
Patient communication and consultation	3.4	3.3	0.6
Patient monitoring and follow-up	3.3	3.1	1.5
Disease prevention, diagnosis and treatment	3.6	3.3	2.1**

** *P* < 0.05; *** *P* < 0.001.

### Measurement model

Component-based SEM was used to empirically test our set of research hypotheses. The PLS technique was selected because it is suited to measurement models such as ours that include both exogenous (reflective) and endogenous (formative) constructs.^50^ Precisely, whereas the research construct “individual background” is modeled as being “formative” given its composite and multidimensional nature, the four other constructs are modeled as being “reflective”.^51^

The initial step consisted of estimating the measurement model at t_0_ and t_1_ using the SmartPLS software. We started by assessing the internal consistency of the measures (manifest variables) as well as the unidimensionality, reliability, predictive validity, and discriminant validity of the four reflective research constructs (latent variables). As shown in Table 4, all the Cronbach α values (except one at t_1_) were above the .70 threshold, thus confirming the internal consistency of the research variables. The unidimensionality of a reflective construct is assessed by looking at each of its indicators’ loading (λ), the threshold being 0.40 for newly developed scales.^52^

**Table 4.** Psychometric properties of the research variables and constructs.

	Pre-COVID-19 (t_0_)				Peri-COVID-19 (t_1_)			
Research construct		CR^b^	AVE^c^			CR^b^	AVE^c^	
Research variable	α^a^			VIF^d^	α^a^			VIF^d^
Individual background		-	-			-	-	
Age (yrs.)	-			1.11	-			1.23
Gender (0: male, 1: female)	-			1.02	-			1.08
Academic level (1 to 5)	-			1.12	-			1.2
Experimentation with dHealth technologies		0,81	0.47			0,81	0.48	
With basic IT systems	0.86			-	0.87			-
With advanced dHealth	0.88			-	0.81			-
With telehealth	0.78			-	0.69			-
With AI-related technologies	0.84			-	0.84			-
With mobile applications	0.88			-	0.86			-
Importance of dHealth in the curriculum		0.90	0.70			0.93	0.76	
On basic IT systems	0.93			-	0.97			-
On advanced dHealth technologies	0.93			-	0.93			-
On telehealth	0.86			-	0.87			-
On AI-related technologies	0.84			-	0.84			-
Role of AI in the future of medicine		0.91	0.76			0.92	0.80	
For the medical profession	0.82			-	0.78	-		-
For the medical specialties	0.82			-	0.79			-
For their medical practice	-			-	-			-
Intention to integrate dHealth in future practice		0.98	0.95			0.99	0.98	
For patient communication and consultation	0.94			-	0.97			-
For patient monitoring and follow-up	0.91			-	0.95			-
For disease prevention, diagnosis and treatment	0.95			-	0.97			-

^a^ Cronbach alpha coefficient of reliability.

^b^ CR: composite reliability. [CR = (Σλ_i_)^2^/((Σλ_i_)^2^ + Σ(1-λ_i_^2^))] .

^c^ AVE: average variance extracted by a construct from its associated variables. [AVE = ∑λ_i_^2^ / n] .

^d^ VIF: variance inflation factor. VIF = 1 / (1 – R_i_^2^), where R_i_^2^ is the unadjusted R^2^ obtained when variable *i* is regressed against all other variables forming a construct.

Moreover, the reflective constructs’ composite reliability (CR) must be above the 0.7 threshold, which is the case for all four reflective constructs. There is also evidence of the convergent validity of these constructs because their average variance extracted (AVE) is above the 0.50 threshold, except for the “Experimentation with dHealth” construct which is just below this threshold (0.47). Despite this, it was decided not to remove any of this last construct's indicators from its measure, given that it demonstrated adequate reliability and unidimensionality. Finally, there is evidence of discriminant validity, that is, of the extent to which a research construct differs from other constructs. In the case of the four reflective constructs, the shared variance between each construct and the other constructs was found to be less than the AVE from its indicators.^52^ In the case of the sole formative construct, individual background, the fact that it shared less than 70% variance with the other construct in the measurement model, and thus correlated less than perfectly with these constructs, was again indicative of strong discriminant validity.^51^

Next, as the usual validity criteria for reflective constructs are inapplicable to a formative construct, one must instead verify that there is no multicollinearity among the formative construct's indicators. One uses the variance inflation factor (VIF) statistic to do so, a common rule being that a variable's VIF value be less than 3.3, or in other words, that less than 70% of the variance in the variable be jointly explained by the other variables.^53^ As shown in Table 4, the outer VIF values estimated by PLS for the three formative indicators of the individual background construct varied from 1.02 to 1.12 at t_0_ and from 1.08 to 1.23 at t_1_, well below the 3.3 threshold, thus indicating the absence of multicollinearity.

Measuring the research variables through a self-administered questionnaire with a single respondent poses a risk of common method bias (CMB).^54^ As precautionary measures, we chose different question formats and scale types. Further, we examined the correlation matrix of the five research constructs to determine if any two constructs correlated above 0.90, as this could signal the presence of CMB in the data.^55^ In our case, all construct correlations (not shown here) were well-below this threshold. We also employed Harman's single-factor test to check for CMB, examining the unrotated factor solution for all variables in the measurement model. As multiple factors emerged from the factor analysis and as no single factor accounted for 50% or more of the covariance among the variables, this further suggests the absence of CMB.^56^

### Causal analysis at t_0_

**Figure 2.**
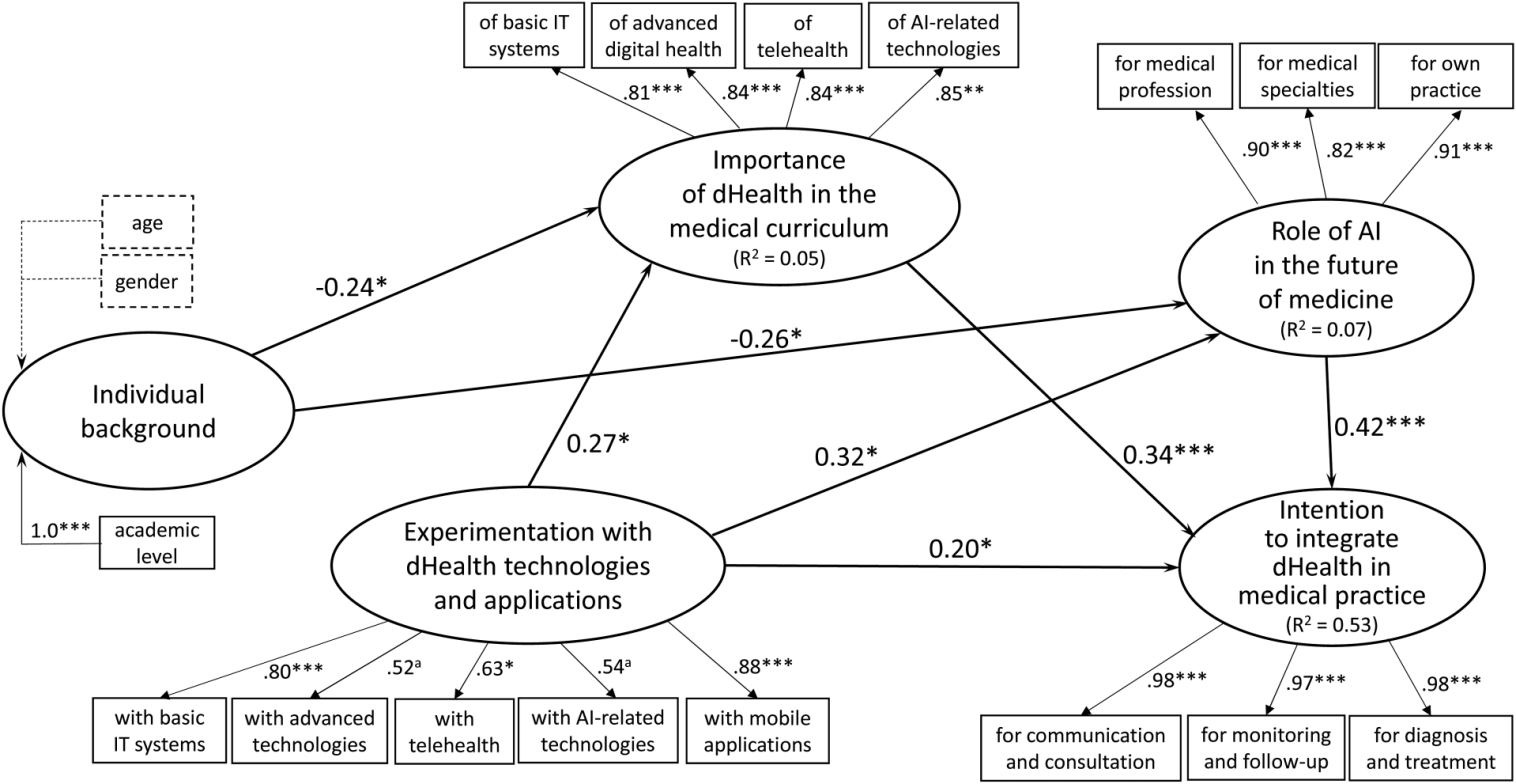
Causal analysis results at t_0_.

Next, the causal paths inferred from the conceptual framework were tested by assessing the path coefficients (β) estimated by the SEM procedure as executed by the SmartPLS software. As shown in Figure 2, the performance of the theoretical model at t_0_ that interrelates the five research constructs is indicated by the strength and significance of the βs and the proportion of explained variance (R^2^ = 0.53), as befits PLS's focus on prediction and concern with generalization.^57^

*Hypothesis 1* (partly confirmed). Given the results of the initial causal analysis (t_0_) provided by the SEM procedure, an initial finding lies in the negative and highly significant path coefficient that links the medical students’ characteristics to their beliefs about the role of AI in the future of medicine (β = −0.26, *P* < 0.05). As shown in Figure 2, the primary explanatory characteristic is the academic level, as students who are in their preparatory and preclinical years have stronger beliefs about the positive impact of AI on the medical profession, various medical specialities, and their own medical practice. Age and gender were not significantly associated with students’ beliefs about AI technologies.

*Hypothesis 2* (partly confirmed). Another related finding is that medical students’ characteristics are negatively and significantly associated to their perceived dHealth education needs (β = −0.24, *P* < 0.05). Here again, the primary explanatory characteristic is the academic level, as students in their preparatory or preclinical years express more important needs than those in their clerkship years. One may surmise that the more advanced students, having experimented more with various dHealth technologies and applications during rotations, are more realistic as to the place of dHealth in the curriculum and thus have less expectations in this regard. Age and gender were not associated with perceived dHealth education needs.

*Hypothesis 3* (confirmed). As expected, we found a positive and significant relationship between medical students’ experimentation with dHealth and their beliefs about the role of AI technologies in the future of medicine and their own medical practice (β = 0.32, *P* < .05).

*Hypothesis 4* (confirmed). Another finding concerns the positive influence of the medical students’ experimentation with dHealth technologies on their dHealth education needs (β = 0.27, *P* < 0.05). In other words, greater exposure to and practical experience with dHealth technologies lead students to understand and appreciate the pivotal role of these technologies in healthcare and the necessity of integrating dHealth into medical education.

*Hypothesis 5* (confirmed). We also found a positive and significant relationship between students’ level of experimentation with dHealth technologies and their intention to integrate dHealth into their practice (β = 0.20, *P* < 0.05). In other words, experimenting with dHealth technologies during their medical degree spurs students’ intention to integrate these systems and tools in their future practice. Now, given that the indirect effects of experimenting with dHealth on the students’ intention (through their dHealth education needs and beliefs about AI) are greater than the direct effects, this finding is rather tentative and calls for further empirical validation.

 *Hypothesis 6* (confirmed). The next finding lies in the positive and highly significant path coefficient (β = 0.42, *P* < 0.001) that links medical students’ beliefs about the impact of AI on medicine to their behavioral intention. This result is in line with Triandis’ interpersonal behavior theory^36^ which postulates that individuals’ behavioral intention is influenced by their beliefs toward the behavior.

 *Hypothesis 7* (confirmed). The study's final finding lies in the positive and significant path coefficient (β = 0.34, *P* < 0.001) that confirms the influence of medical students’ dHealth education needs on their intention to integrate dHealth in their practice. This finding is in line with prior TAM^41^ and UTAUT (unified theory of acceptance and use of technology) research.^58^

### Causal analysis at t_1_

**Figure 3.**
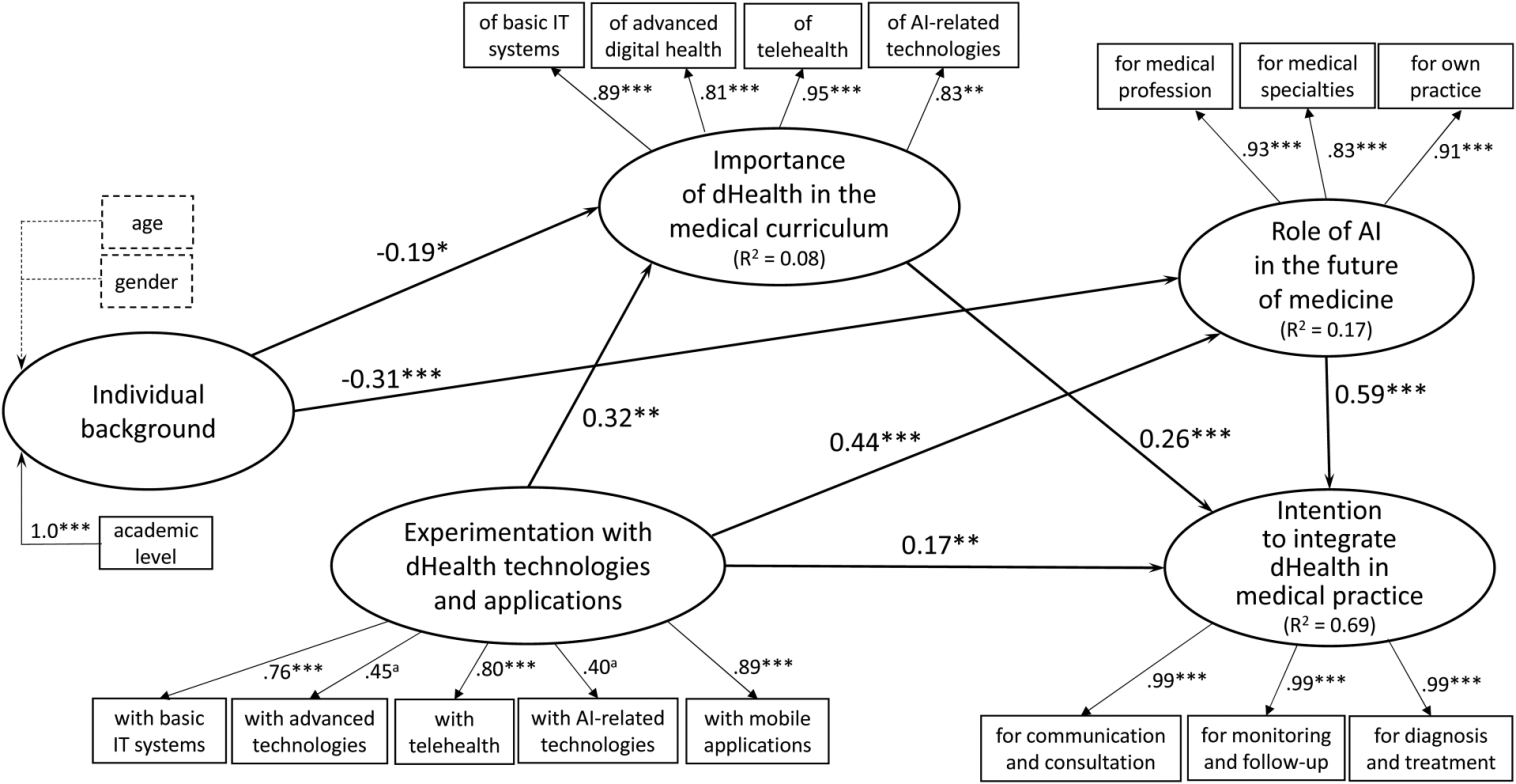
Causal analysis results at t_1_.

As depicted in Figure 3, the causal paths inferred from the research model were tested anew at t_1_ by assessing the path coefficients (β) estimated by the SEM procedure. As indicated by the strength and significance of the βs and the proportion of explained variance, the performance of the theoretical model was found in the replication study to be superior to that of the initial study (R^2^_t0_ = 0.53 vs. R^2^_t1_ = 0.69). These results provide further confirmation of the validity and predictive ability of our theoretical model. Moreover, the advent of the COVID-19 pandemic appears to have had no impact on the theorized relationships between the research constructs, as replicating the causal analysis produced results similar to those observed at t_0_, that is, in terms of the strength and significance of these relationships. The reader may thus essentially refer to the above presentation of the causal analysis at t_0_ for further analysis of the t_1_ results.

## Discussion

### Principal findings

The present study provides a clear and concise explanation of medical students’ intention to integrate dHealth technologies into their practice. We found the sampled students, especially those who are in their preparatory or preclinical years, to have had little occasion to experiment with dHealth technologies during their medical studies. A plausible explanation might be that during their preclinical years, students do not have direct access to EMRs and other clinical information systems available in hospital settings. The lower scores at t_1_ might be related to the pandemic where students were even less exposed to hospital practice (and hospital IT systems), with many parts of the curriculum being offered virtually.

This lack of exposure may explain why a vast majority of respondents felt that all medical students should receive dHealth training as part of their formal education. Further, most had strong beliefs about the positive impact of AI-related technologies in the future of medicine, in general, and the progress of various medical disciplines. Importantly, a majority of students declared an intention to integrate dHealth technologies, including AI-based tools, into their medical practice.

The main differences observed between t_0_ and t_1_ brings telehealth, and teleconsultation in particular, to the forefront of medical education. Indeed, the pandemic's advent appears to have shifted medical students’ view of telemedicine, now considered to be an important aspect of their dHealth education and future medical practice. Moreover, this shift is made at the expense of another component, basic IT systems (eg, EMRs), that could be now considered as a ‘given’,^59^ that is, as being fully and seamlessly integrated within medical education and medical practice.

Last, as predicted by our theoretical model, the combination of three factors (experimentation with dHealth, perceived importance of integrating dHealth into medical curriculum, and beliefs about AI in medicine) explains, in both phases of the study, medical students’ intention to integrate dHealth into their practice.

### Study contributions and implications

The factors included in the theoretical model constitute important descriptive, predictive, and explanatory keys upon which to reflect on the issue of dHealth education in medical schools. The proposed model may thus constitute an initial conceptual framework for researchers and practitioners concerned with informing, motivating, and preparing medical students to make effective use of dHealth technologies, in general, and of AI tools, in particular. In this regard, it appears that the emphasis on basic IT systems such as EMRs still remains, whereas telehealth appears to have taken on added importance with the advent of the COVID-19 pandemic. Furthermore, as the students’ future use of dHealth technologies is determined above all by their beliefs about the positive impact of AI on modern medicine, it ensues that the introduction of AI training in medical curricula should be further considered and investigated.^60^

This study has several practical implications. For one thing, our study provides medical schools with a conceptual template with which to make a strategic assessment of their dHealth situation and thus obtain actionable insights as to the technologies to be included in the medical curriculum. Our findings also allow us to make a few recommendations to medical schools in Canada and elsewhere wishing to better serve their students through the mindful integration of dHealth within the curriculum: (1) integrate dHealth training as part of the doctorate in medicine; (2) create one or several specialized diplomas in dHealth (eg, telehealth, AI in health); (3) set-up a working group within each faculty of medicine, comprised of professors and students of all levels, to periodically review dHealth training needs in light of existing and emerging technologies; (4) periodically assess students’ satisfaction with their dHealth training, and make the necessary adjustments; and (5) foster collaboration and exchange between medical students and those from other faculties (eg, computer science, software engineering) interested in dHealth technologies through the organization of “hacking health” type of events.

Importantly, our results have implications for key institutional actors in the development and deployment of dHealth technologies in Canada, as they seek to guide and support medical schools in their integration of these technologies within the medical curriculum. For instance, the Association of Faculties of Medicine of Canada (AFMC) and the Canadian Medical Association (CMA) could collaborate on developing and implementing an evidence standards framework for dHealth technologies, like the one developed in the United Kingdom by the National Institute for Health and Care Excellence.^61^ This framework would enable a more agile evaluation of these technologies in medical school settings, as well as render them more meaningful to students and add value to dHealth education.

### Study limitations and suggestions for future research

The results of this study must be interpreted with caution due to its inherent limitations. Given the nature of the sample, its representativeness in relation to all Canadian medical students limits the scope of these results. Future research could investigate the nature and scope of dHealth education offered in medical schools located elsewhere in Canada as well as in other countries and compare students’ level of proficiency in dHealth as well as their intention to integrate dHealth technologies in their medical practice. It would also be important to conduct a follow-up survey once the COVID-19 pandemic is behind us. Moreover, the rather low response rate may have induced a non-response bias that is hard to evaluate.^62^ Notwithstanding our aim for parsimony, the theoretical model could also be extended in future research by including other variables such as social influence and effort expectancy to be more comparable to the previously cited behavioral studies. Further, one could include, in addition to dHealth technologies and applications, the IT-enabled medical knowledge management capabilities such as e-healthcare intelligence and e-collaboration that students must develop to practice modern medicine and, hence, be both innovative and productive.^63,64^

## Conclusions

This study provides a better understanding of the factors predicting medical students’ intention to integrate dHealth into their medical practice. Based upon a multi-theory behavioral model, it reveals the specific dHealth technologies and applications that could be inserted in the medical curriculum to encourage and facilitate students’ adoption of these technologies. Medical schools and faculties are asked to do more and better about preparing their students for the effective use of dHealth in their medical practice. It thus behooves them to develop their dHealth education resources and capabilities in coherence with this imperative.

## Notes

1. Facts and Factors (2021). Available at: https://www.globenewswire.com/en/news-release/2021/09/02/2290548/0/en/Global-Report-on-Digital-Health-Market-Size-Valuation-Will-Reach-to-USD-220-94-Billion-by-2026-According-to-Facts-Factors.html.

2. See https://www.canada.ca/en/health-canada/services/drugs-health-products/medical-devices/activities/announcements/notice-digital-health-technologies.html and https://www.fda.gov/medical-devices/digital-health.

3. Called INESSS.

## Appendix I. Questionnaire content and descriptive statistics.

[Table table5-20552076221114195] [Table table6-20552076221114195] [Table table7-20552076221114195] [Table table8-20552076221114195]

**Table A1. table5-20552076221114195:** Level of experimentation with dHealth technologies.

dHealth technology bundle	dHealth technologies and applications	Pre-COVID-19 (t_0_)				Peri-COVID-19 (t_1_)			
		1	2 or 3	4 or 5	Mean	1	2 or 3	4 or 5	Mean
Basic IT systems^a^	Interoperable electronic health record (iEHR) system^c^	48%	33%	19%	2.2	62%	31%	7%	1.6
	Electronic medical record (EMR) systems	57%	23%	23%	2	72%	20%	8%	1.5
	Clinical information systems (CIS)	56%	23%	29%	1.9	75%	21%	4%	1.4
	Interoperable personal health record (iPHR) system^d^	71%	26%	26%	1.4	79%	20%	1%	1.3
	Interoperable medical appointment system (iMAS)^e^	78%	19%	19%	1.3	83%	16%	1%	1.2
TeleHealth^a^	Teleconsultation	83%	17%	0%	1.2	54%	43%	3%	1.7
	Tele-expertise	90%	9%	1%	1.1	80%	18%	2%	1.3
AI-related technologies^a^	Artificial intelligence	71%	27%	2%	1.4	80%	18%	2%	1.3
	Machine learning	86%	13%	1%	1.2	90%	9%	1%	1.1
	Big data in healthcare	86%	13%	1%	1.2	93%	7%	0%	1.1
Advanced dHealth^a^	Robotics in healthcare	69%	30%	1%	1.4	81%	18%	1%	1.2
	Virtual reality	84%	13%	3%	1.3	88%	12%	0%	1.1
	Nanotechnology	86%	13%	1%	1.2	91%	9%	0%	1.1
	Augmented reality	87%	11%	2%	1.2	91%	9%	0%	1.1
	3D printing	83%	16%	1%	1.2	88%	12%	0%	1.1
	Internet of things	90%	9%	1%	1.1	94%	5%	1%	1.1
	Blockchain	91%	8%	1%	1.1	96%	4%	0%	1
Mobile applications^b^	UpToDate	48%	20%	32%	2.5	57%	27%	16%	1.9
	INESSS (Quebec HTA Institute's mobile app)	42%	36%	22%	2.3	51%	35%	14%	2
	Medscape	52%	36%	12%	1.9	67%	27%	6%	1.6
	Lanthier	70%	15%	15%	1.7	86%	8%	6%	1.3
	MedCalc	73%	19%	8%	1.6	84%	14%	2%	1.2
	BMJBestPractice	75%	19%	6%	1.5	83%	15%	2%	1.3

^a^ 5-point scales [1 = never exposed to the technology, 2, 3 = somewhat exposed, 4, 5 = very exposed].

^b^ 5-point scales [1 = application never used, 2, 3 = used rarely or regularly, 4, 5 = used often or very often] – Only the top-6 apps are shown here.

^c^ Named *« Dossier Santé Québec »* in French.

^d^ Named *« Carnet Santé Québec »* in French.

^e^ Named *« Rendez-vous Santé Québec »* in French.

**Table A2. table6-20552076221114195:** Perceived importance of dHealth in the medical curriculum.

dHealth technology bundle	dHealth technologies	Pre-COVID-19 (t_0_)				Peri-COVID-19 (t_1_)			
		1	2 or 3	4 or 5	Mean	1	2 or 3	4 or 5	Mean
Basic IT systems^a^	Interoperable electronic health record (iEHR) system^b^	0%	24%	76%	4.2	0%	33%	67%	4.1
	Electronic medical record (EMR) systems	0%	24%	76%	4.2	0%	31%	69%	4.1
	Clinical information systems (CIS)	1%	27%	72%	4.1	0%	37%	63%	4
	Interoperable personal health record (iPHR) system^b^	1%	32%	67%	3.9	0%	36%	64%	4
	Interoperable medical appointment system (iMAS)^d^	0%	27%	73%	4	0%	35%	65%	4
TeleHealth^a^	Teleconsultation	2%	43%	55%	3.7	0%	29%	71%	4.2
	Tele-expertise	2%	51%	47%	3.6	0%	35%	65%	4
AI-related technologies^a^	Artificial intelligence	1%	43%	56%	3.7	1%	48%	51%	3.6
	Machine learning	2%	57%	41%	3.5	1%	59%	40%	3.5
	Big data in healthcare	3%	65%	32%	3.4	1%	68%	31%	3.4
Advanced technologies^a^	Robotics	1%	45%	54%	3.7	1%	43%	56%	3.8
	virtual reality	1%	63%	36%	3.4	2%	67%	31%	3.3
	Nanotechnology	3%	60%	37%	3.4	3%	64%	33%	3.4
	Augmented reality	2%	65%	33%	3.3	2%	67%	31%	3.3
	3D printing	4%	62%	34%	3.3	1%	63%	36%	3.4
	Internet of things	2%	70%	28%	3.3	2%	73%	25%	3.1
	Blockchain	5%	73%	22%	3.1	4%	76%	20%	3.1

^a^ 5-point Likert scales [1 = totally disagree, 2, 3 = neither disagree nor agree, 4, 5 = totally agree].

^b^ Named *« Dossier Santé Québec »* in French.

^c^ Named *« Carnet Santé Québec »* in French.

^d^ Named *« Rendez-vous Santé Québec »* in French.

**Table A3. table7-20552076221114195:** Beliefs about the impact of AI on medicine.

		Pre-COVID-19 (t_0_)				Peri-COVID-19 (t_1_)			
		1	2 or 3	4 or 5	Mean	1	2 or 3	4 or 5	Mean
On the medical profession^a^	Prevention of diseases	0%	42%	58%	3.7	0%	47%	53%	3.6
	Diagnosis of diseases	1%	29%	70%	3.9	0%	41%	59%	3.7
	Treatment of diseases	0%	39%	61%	3.8	0%	45%	55%	3.7
	Prognosis of diseases	0%	45%	55%	3.7	0%	51%	49%	3.6
	Doctor-patient relationship	8%	80%	12%	2.7	2%	85%	13%	2.8
On the medical specialties^b^	Anatomopathology	1%	46%	53%	3.7	0%	54%	46%	3.7
	Radiology	0%	32%	68%	4.2	0%	41%	59%	4
	Dermatology	2%	59%	39%	3.4	0%	70%	30%	3.3
	Ophthalmology	1%	49%	50%	3.6	1%	58%	41%	3.5
	Urgent care and critical care	1%	67%	32%	3.3	1%	68%	31%	3.2
	Family medicine	2%	67%	31%	3.2	1%	75%	24%	3.2
	Internal medicine	1%	59%	40%	3.4	0%	66%	34%	3.3
	Psychiatry	13%	78%	9%	2.5	7%	30%	63%	2.6
	Surgery	0%	54%	46%	3.6	1%	56%	43%	3.6
		1		2		1		2	
On medical students’ future practice^c^	Analyze radiological images	89%		11%		97%		3%	
	Analyze photographical images	87%		13%		91%		9%	
	Analyze pathological images	84%		16%		86%		14%	
	Make diagnoses with regard to patients	59%		41%		67%		33%	
	Make prognoses with regards to patients	70%		30%		87%		13%	
	Determine patient care protocols	74%		26%		76%		24%	
	Analyze anamnesis data and form a medical opinion	55%		45%		57%		43%	
	Supervise and evaluate exchanges with patients	29%		71%		34%		66%	

^a^ 5-point scales [1 = very negative effect of AI and ML, 2, 3 = slight negative or no effect, 4, 5 = positive or very positive effect].

^b^ 5-point scales [1 = unaffected by AI and ML, 2, 3 = uncertain, 4, 5 = affected or very affected by AI and ML].

^c^ 2-point-scales [1 = yes, 2 = no].

**Table A4. table8-20552076221114195:** Intention to integrate dHealth in one's own medical practice.

		Pre-COVID-19 (t_0_)				Peri-COVID-19 (t_1_)			
		1	2 or 3	4 or 5	Mean	1	2 or 3	4 or 5	Mean
For patient communication and consultation^a^	Use an online medical appointment platform	20%	10%	70%	3.7	32%	9%	59%	3.4
	Do virtual consultations with patients	22%	52%	26%	2.7	32%	9%	59%	3.2
	Communicate by email or SMS with patients	22%	36%	42%	3	34%	20%	46%	2.9
	Communicate by email or SMS with other physicians	17%	3%	78%	4	32%	1%	67%	3.6
For patient monitoring and follow-up^a^	Prescribe mobile applications to patients	20%	28%	52%	3.2	33%	21%	46%	2.9
	Recommend reliable and secure websites to patients	20%	7%	73%	3.8	32%	22%	46%	3.5
	Prescribe connected medical devices (objects) to patients	21%	41%	38%	3.8	34%	27%	39%	2.8
	Use smart medical devices to assess patients’ health	21%	28%	51%	3.3	33%	13%	54%	3.1
For disease prevention, diagnosis and treatment^a^	Solicit second opinions via a tele-expertise platform	19%	18%	63%	3.5	32%	9%	59%	3.3
	Consult medical websites to assist me in my practice	17%	1%	80%	4.1	32%	1%	67%	3.6
	Use mobile applications to assist me in my practice	19%	3%	78%	4	31%	20%	49%	3.5
	Use systems based on AI algorithms to assist me in my practice	20%	33%	47%	3.2	33%	23%	44%	2.9

^a^ 5-point scales [1 = very improbable, 2 = improbable, 3 = uncertain, 4 = probable, 5 = very probable].
